# A Rare Pediatric Case of COVID-19-Associated Guillain-Barré Syndrome With Multiple Cranial Neuropathies and Without Pulmonary Symptoms

**DOI:** 10.7759/cureus.75930

**Published:** 2024-12-18

**Authors:** Yavuz Atas, Serkan Kirik, Hatice G Poyrazoglu, Yesim Eroglu, Zülal A Toraman

**Affiliations:** 1 Department of Pediatrics, Division of Pediatric Neurology, Fırat University School of Medicine, Elazıg, TUR; 2 Department of Pediatrics, Division of Pediatric Neurology, Fırat University School of Medicine, Elazig, TUR; 3 Department of Radiology, Firat University School of Medicine, Elazig, TUR; 4 Department of Microbiology, Fırat University School of Medicine, Elazig, TUR

**Keywords:** cranial nerves, guillain-barré syndrome, pediatric, polyneuropathy, sars-cov-2

## Abstract

Coronavirus disease (COVID-19) is a disease caused by severe acute respiratory syndrome coronavirus 2 (SARS-CoV-2) that primarily affects the respiratory system but can also lead to neurological complications such as Guillain-Barré syndrome (GBS). This case report describes an eight-year-old boy with COVID-19-associated GBS involving multiple cranial nerves (third, seventh, and ninth) without pulmonary symptoms. The patient initially presented with flu-like symptoms along with right facial paralysis, which progressed to bilateral facial paralysis, limb weakness, and sensory loss. Neurologic examination revealed a loss of deep tendon reflexes, while cerebrospinal fluid analysis showed albuminocytologic dissociation. The SARS-CoV-2 polymerase chain reaction (PCR) test was positive in the nasopharyngeal swab but negative in the cerebrospinal fluid. The patient was treated with intravenous immunoglobulin (IVIG) and showed marked improvement, regaining the ability to walk unassisted within a week. This case highlights the neuroinvasive potential of SARS-CoV-2 and demonstrates that COVID-19 in pediatric patients can be associated with neurological complications such as GBS, even without respiratory symptoms.

## Introduction

Novel coronavirus disease (COVID-19) is an infectious disease with a global impact caused by a type of coronavirus known as severe acute respiratory syndrome coronavirus 2 (SARS-CoV-2). Pneumonia is the most common clinical presentation among patients with SARS-CoV-2 infection, sometimes leading to acute severe respiratory distress syndrome (ARDS) requiring admission to the intensive care unit [1-3].

There is an increasing body of evidence suggesting that coronaviruses may cause neurological disorders by affecting the central nervous system (CNS); however, there is limited information about the prognosis and treatment of neurological involvements associated with SARS-CoV-2 in pediatric patients [1-3]. Characteristics and pathogenesis of the neurological complications of COVID-19 should be clarified. The most emphasized theory is that considering that ACE2 is found in capillary endothelium, SARS-CoV-2 may cause blood-brain barrier injury and reach the central nervous system by means of ACE2 [2, 4-6]. In addition, severe neurological conditions such as Guillain-Barré syndrome (GBS) associated with COVID-19 may also rarely occur [7]. Therefore, it is important for clinicians to be aware of these neuroinvasive properties of SARS-CoV-2 when caring for COVID-19 patients. Guillain-Barré syndrome is an acute/subacute onset polyradiculoneuropathy that typically develops within a few days with sensory symptoms and weakness and usually causes quadriparesis; it manifests hyporeflexia and may be accompanied by cranial neuropathies [5]. Nearly 70% of patients have a history of a recent upper or lower respiratory infection or a gastrointestinal disease [1]. The patient's subclinical complaints were observed with COVID-19 parainfection, and the GBS clinic became evident in the postinfectious period [6,7]. Most human viruses, including coronaviruses, possess tropism and neuronal invasion characteristics, which have the potential to cause other disorders. Additionally, other studies on the inflammatory aspect of the cytokine storm attempt to explain the neurological symptoms of COVID-19 as resulting from inflammatory cascades, namely the presence of a cytokine storm, thereby exploring the relationship between COVID-19 and GBS. Due to the inflammatory mechanism caused by SARS-CoV-2, it actively contributes to the increase in inflammatory response and the insufficiency of control mechanisms in the immune system. Thus, it can lead to GBS and other neurological diseases [6,7]. 

We report a pediatric SARS-CoV-2-associated GBS case that started with peripheral facial paralysis and progressed to multiple cranial neuropathy and progressive limb weakness.

## Case presentation

An eight-year-old boy was presented to our outpatient clinic with rhinorrhea, a fever of 38.2°C, and right peripheral facial paralysis that started five days ago. No vaccination history before the symptoms. Other systemic examinations were normal. He was sent home with symptomatic treatment with eye drops and physical therapy recommendations. Four days after his discharge, in June 2020, he was readmitted to the hospital with a preliminary diagnosis of acute flaccid paralysis after presenting with bilateral facial weakness, inability to smell and taste, diffuse myalgia in both lower and upper extremities, and weakness that had progressively worsened within the last 24 hours. He didn't require intensive care unit admission. He had a blood pressure of 107/68 mmHg, an oxygen saturation of 96%, and a body temperature of 37.6°C. He was free of altered consciousness.

His neurological examination was notable for the inability to stand upright without assistance and difficulty walking, bilateral facial paralysis, and absence of pharyngeal reflex. His deep tendon reflexes were absent in all four extremities. His motor strength was rated 4/5 in both lower extremities and 4/5 in both upper extremities. He also had a tingling sensation and a loss of touch sensation.

**Figure 1:**
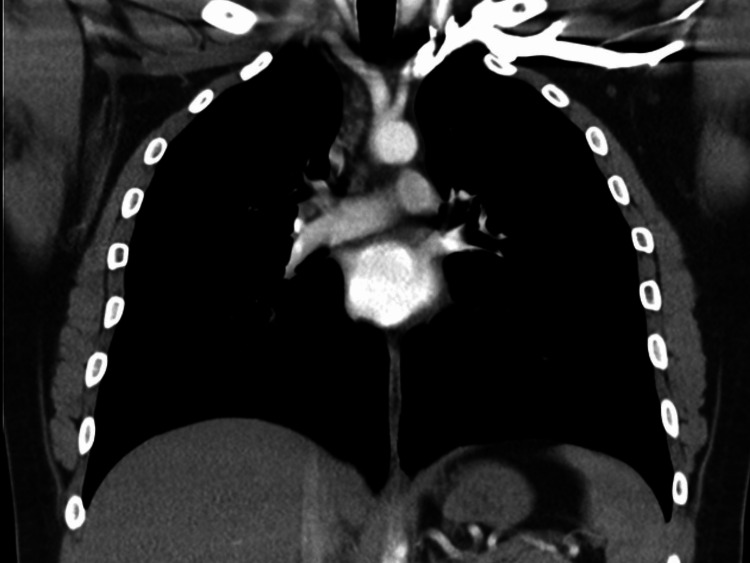
Normal CT image of the chest

**Figure 2:**
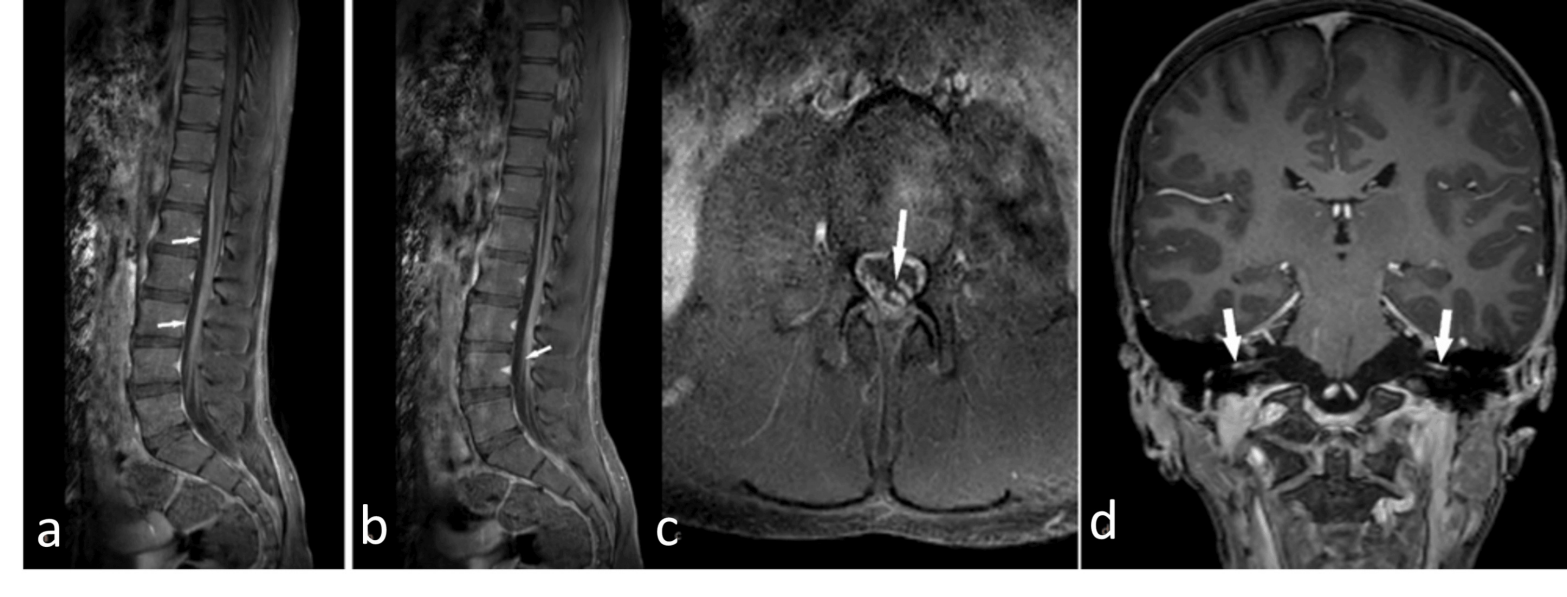
MRI examination of the patient at administration Contrast-enhanced MRI findings in cauda equina and bilateral facial neuritis Fat-suppressed, contrast-enhanced sagittal T1-weighted lumbar MRI shows marked contrast enhancement of thickened nerve roots of anterior (a) and posterior (b) cauda equina (white arrows). Fat-suppressed axial T1-weighted MRI shows marked contrast enhancement of thickened nerve roots of the cauda equina (c). Contrast-enhanced coronal T1-weighted brain MRI shows enhancement consistent with bilateral facial neuritis within the bilateral internal acoustic canals (d).

In the laboratory analysis, complete blood count, creatine kinase, liver and urine function tests, electrolyte levels, D-dimer level, vitamin B12, folic acid, thyroid function tests, anti-thyroglobulin, anti-nuclear antibody, and ferritin levels were normal. There were mild elevations in the fibrinogen level (501.8 mg/dL; normal range 190-350) and C-reactive protein level (8.9 mg/dL; normal range < 5 mg/dL). Ganglioside antibody panel tests were in normal ranges. The CT scan of the chest was normal (Figure 1). Cranial and spinal magnetic resonance imaging (MRI) examination revealed thickening of the cauda equina fibers at the level of the conus medullaris and significant contrast enhancement in the nerve roots and facial nerve following contrast medium administration (Figure 2).

A lumbar puncture was performed on the first day of hospitalization; the cerebrospinal fluid (CSF) chemistry test showed a normal glucose level of 47 mg/dL (normal range: 40-70 mg/dL) and an elevated protein level of 160.7 mg/dL (normal range: 15-45 mg/dL). The CSF contained 6 cells/μL (83.3% lymphocyte). These findings were considered in favor of albuminocytological dissociation.

A respiratory virus panel by multiplex polymerase chain reaction (PCR) test was performed. 2019-nCoV PCR was found positive. Influenza A-B virus, influenza A (H1N1) virus, human rhinovirus, human coronavirus NL63-229E-OC43-HKU1, human parainfluenza 1-2-3-4, human metapneumoviruses A/B, human bocavirus, *Mycoplasma pneumoniae*, human respiratory syncytial viruses A/B, human adenovirus, enterovirus, *Legionella pneumophila*, *Bordetella* spp., and influenza A H1 PCR tests were all negative. In the CSF examination, PCR tests and cultures, including herpes virus, varicella-zoster virus, *Streptococcus pneumoniae*, *Neisseria meningitides*, *Mycoplasma pneumonia*, and *Mycobacterium tuberculosis*, were normal. The SARS-CoV-2 PCR test from a CSF sample was reported negative. The tests for *Brucella *and Lyme disease yielded normal results. A stool examination revealed no cells, and no microorganisms, including *Campylobacter jejuni*, rotavirus, or adenovirus, were detected.

**Figure 3:**
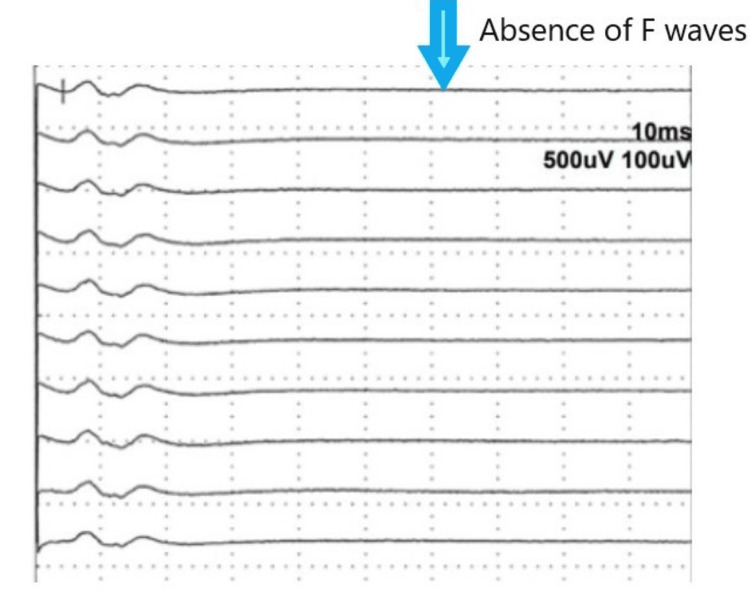
The neurological examination performed at admission showed an absence of F waves (blue arrow).

An electromyography (EMG) revealed no F-wave responses (Figure 3). The patient was diagnosed with COVID-19-associated GBS with polycranial nerve involvement. On the first day of hospitalization, he was begun on intravenous immunoglobulin (IVIG) infusions at a dose of 0.4 g/kg/day for five days. His signs and symptoms showed signs of improvement on the fourth day of admission. He began walking without support on the seventh day. Since a marked improvement was observed in walking ability and weakness on the 12^th^ day, he was discharged with the recommendations of home isolation and physical therapy and rehabilitation. He could walk without support at the first month of follow-up. His facial paralysis also improved. A gag reflex could be elicited. His deep tendon reflexes were diminished. In control EMG, recovery to normal was observed in electrophysiological findings, including F-wave responses.

## Discussion

In GBS, a spectrum of polyneuropathies is characterized by acutely progressive weakness (developing within one to four weeks), mild-to-moderate sensory abnormalities, cranial nerve involvement, and muscular or radicular pain. Seventy percent of patients with GBS report a history of flu-like infection one to three months prior to presentation. It is one of the autoimmune neurological diseases triggered by viral infections such as influenza, enteroviruses, H1N1, West Nile virus, Zika, Middle East respiratory syndrome coronavirus (MERS-CoV), and SARS-CoV [1,4,6,7]. Toscano et al. reported a case series in which all patients experienced lower and upper extremity weakness, distal paresthesia, facial palsy, and sensory loss five to 10 days after symptoms like cough, anosmia, and sore throat. Although the CSF sample contained a high protein concentration, the CSF COVID-19 PCR test was negative in all cases. The EMG examination showed demyelination and axonal GBS [8]. Khalifa et al. reported that a nasopharyngeal swab PCR test detected SARS-CoV-2, but CSF SARS-CoV-2 PCR was reported negative in a pediatric COVID-19-associated GBS case in an 11-year-old boy [1]. We also report a pediatric case that started with Bell's palsy and flu-like symptoms, which were followed by typical signs of GBS. The emergence of facial weakness, together with the initial symptoms of the disease and the progression of these signs and symptoms to GBS, provides novel information about COVID-19. Considering the rare relationship between (idiopathic or parainfectious) GBS and Bell's palsy, especially in children, the importance of our study becomes evident.

Severe acute respiratory syndrome coronavirus 2 has only recently been identified, yet evidence indicates that neurotropism is a common feature of coronavirus infections. The pathogenesis of cranial neuropathy in SARS-CoV-2 infection may result from immune mechanisms or directly from the virus's neuropathogenic effects [2,9-11]. Animal models have demonstrated that SARS-CoV may probably invade the brain through the olfactory nerve and then rapidly disseminate to certain cerebral regions, including the thalamus and brain stem [11]. Roussel et al. reported the case of a pediatric patient with COVID-19-associated involvement of the fifth, seventh, and ninth cranial nerves [12]. In this study, to the best of our knowledge, our case presentation is the first description of a GBS with cranial polyneuropathy (third, seventh, and ninth) in a child infected with SARS-CoV-2. It is very important that seventh cranial neuropathy was the first neurological sign in our COVID-19-associated GBS case despite the fact that loss of smell, that is, olfactory nerve dysfunction, is usually one of the first neurological signs.

There is increasing evidence that cranial nerve involvement in COVID-19 represents autoimmunity, as in cases of GBS. The virus was not detected in the CSF in seven of 11 patients diagnosed with COVID-19-associated GBS, which explains that a direct root infection or intrathecal viral replication may not take place [9]. Toscano et al. reported that signs of GBS appeared after a period as short as five days when signs of COVID-19 infection persisted, which suggests a parainfectious GBS [7]. In addition, patients showing clinical improvement with IVIG treatment, as with typical GBS patients, support an immune response-related pathogenesis [8,9]. Our patient also developed cranial neuropathy and GBS after moderate fever and facial paralysis but without an antecedent pulmonary involvement. We believe that raising awareness of extrapulmonary clinical findings is crucial in the COVID-19 presentation, where pulmonary symptoms predominate. The single-center nature of our study was the most significant limitation, as it did not include a larger number of cases and diverse clinical features. Therefore, we believe that future studies combining COVID-19-associated GBS cases from multiple centers will contribute to the management of childhood GBS cases associated with COVID-19. This was considered an important and supportive finding to aid in the follow-up of similar cases, which are very limited in number and do not yet have any confirmed therapy.

## Conclusions

This case report is the first that describes pediatric GBS presenting with SARS-CoV-2 infection associated with multiple cranial neuropathies (third, seventh, and ninth) without pulmonary involvement. Our study is of importance in terms of neurological signs associated with SARS-CoV-2, many aspects of which are still unclear. Although COVID-19 is known to cause a milder course in children than adults, the mild flu-like symptoms and the progressive facial palsy leading to a serious neuromuscular condition such as GBS in our patient are of great importance for both pediatricians and pediatric neurologists.

## References

[1] Khalifa M, Zakaria F, Ragab Y, Saad A, Bamaga A, Emad Y, Rasker JJ (2020). Guillain-Barré syndrome associated with severe acute respiratory syndrome coronavirus 2 detection and coronavirus disease 2019 in a child. J Pediatric Infect Dis Soc.

[2] Baig AM, Khaleeq A, Ali U, Syeda H (2020). Evidence of the COVID-19 virus targeting the CNS: tissue distribution, host-virus interaction, and proposed neurotropic mechanisms. ACS Chem Neurosci.

[3] Yeh EA, Collins A, Cohen ME, Duffner PK, Faden H (2004). Detection of coronavirus in the central nervous system of a child with acute disseminated encephalomyelitis. Pediatrics.

[4] Das G, Mukherjee N, Ghosh S (2020). Neurological insights of COVID-19 pandemic. ACS Chem Neurosci.

[5] Saheb Sharif-Askari N, Saheb Sharif-Askari F, Alabed M, Temsah MH, Al Heialy S, Hamid Q, Halwani R (2020). Airways expression of SARS-CoV-2 receptor, ACE2, and TMPRSS2 is lower in children than adults and increases with smoking and COPD. Mol Ther Methods Clin Dev.

[6] Christy A (2020). COVID-19: a review for the pediatric neurologist. J Child Neurol.

[7] Zhao H, Shen D, Zhou H, Liu J, Chen S (2020). Guillain-Barré syndrome associated with SARS-CoV-2 infection: causality or coincidence?. Lancet Neurol.

[8] Toscano G, Palmerini F, Ravaglia S (2020). Guillain-Barré syndrome associated with SARS-CoV-2. N Engl J Med.

[9] Costello F, Dalakas MC (2020). Cranial neuropathies and COVID-19: neurotropism and autoimmunity. Neurology.

[10] Dalakas MC (2020). Guillain-Barré syndrome: the first documented COVID-19-triggered autoimmune neurologic disease: more to come with myositis in the offing. Neurol Neuroimmunol Neuroinflamm.

[11] Gutiérrez-Ortiz C, Méndez-Guerrero A, Rodrigo-Rey S (2020). Miller Fisher syndrome and polyneuritis cranialis in COVID-19. Neurology.

[12] Roussel A, Germanaud D, Bouchoucha Y, Ouldali N, Vedrenne-Cloquet M, Castelle M, Baruchel A (2021). Cranial polyneuropathy as the first manifestation of a severe COVID-19 in a child. Pediatr Blood Cancer.

